# Two closely related 2-(benzo­furan-2-yl)-2-oxoethyl benzoates: structural differences and C—H⋯O hydrogen-bonded supra­molecular assemblies

**DOI:** 10.1107/S2056989017009422

**Published:** 2017-06-30

**Authors:** Li Yee Then, C. S. Chidan Kumar, Huey Chong Kwong, Yip-Foo Win, Siau Hui Mah, Ching Kheng Quah, S. Naveen, Ismail Warad

**Affiliations:** a X-ray Crystallography Unit, School of Physics, Universiti Sains Malaysia, 11800 USM, Penang, Malaysia; b Department of Engineering Chemistry, Vidya Vikas Institute of Engineering & Technology, Visvesvaraya Technological University, Alanahally, Mysuru 570 028, Karnataka, India; c School of Chemical Sciences, Universiti Sains Malaysia, Penang 11800 USM, Malaysia; d Department of Chemical Science, Faculty of Science, Universiti Tunku Abdul Rahman, Perak Campus, Jalan Universiti, Bandar Barat, Perak, Malaysia; e School of Biosciences, Taylor’s University, Lakeside Campus, 47500 Subang Jaya, Selangor, Malaysia; f Institution of Excellence, University of Mysore, Manasagangotri, Mysuru 570 006, India; g Department of Chemistry, Science College, An-Najah National University, PO Box 7, Nablus, West Bank, Palestinian Territories

**Keywords:** crystal structure, benzo­furan, intra­molecular inter­action, inter­molecular inter­action, functional group

## Abstract

The compounds 2-(1-benzo­furan-2-yl)-2-oxoethyl 2-nitro­benzoate, C_17_H_11_NO_6_ (I), and 2-(1-benzo­furan-2-yl)-2-oxoethyl 2-amino­benzoate, C_17_H_13_NO_4_ (II), were synthesized under mild conditions. Their mol­ecular structures were characterized by both spectroscopic and single-crystal X-ray diffraction analysis. The mol­ecular conformations of both title compounds are generally similar. However, different *ortho*-substituted moieties at the phenyl ring of the two compounds cause deviations in the torsion angles between the carbonyl group and the attached phenyl ring. In compound (I), the *ortho*-nitro­phenyl ring is twisted away from the adjacent carbonyl group whereas in compound (II), the *ortho*-amino­phenyl ring is almost co-planar with the carbonyl group. In the crystal of compound (I), two C—H⋯O hydrogen bonds link the mol­ecules into chains propagating along the *c-*axis direction and the chains are inter­digitated, forming sheets parallel to [20-1]. Conversely, pairs of N—H⋯O hydrogen bonds in compound (II) link inversion-related mol­ecules into dimers, which are further extended by C—H⋯O hydrogen bonds into dimer chains. These chains are inter­connected by π–π inter­actions involving the furan rings, forming sheets parallel to the *ac* plane.

## Abstract 2

The title compounds contain a benzo­furan ring and an *ortho*-substituted phenyl ring connected by a carbonyl bridge. The mol­ecular conformations of both compounds are similar, but differ in the torsion angles between the *ortho*-substituted phenyl ring and its adjacent carbonyl group. The crystal structures feature C—H⋯O hydrogen bonds.

## Chemical context   

Oxygen-containing heterocycles are the basic cores of many bioactive structures. Among these, benzo­furan and its derivatives occur frequently in nature because of their stability and ease of generation. Those with substitution(s) at their C-2 and/or C-3 positions are important. Important biological activity such as anti­cancer (Swamy *et al.*, 2015 ▸), anti-acetyl­cholinesterase (Zhou *et al.*, 2010 ▸), anti­microbial (Ugale *et al.*, 2012 ▸) and anti­oxidant (Naik *et al.*, 2013 ▸) actions exhibited by this scaffold have attracted the attention of synthetic chemists. Some of the biological and medicinal significance of benzo­furan derivatives (Nevagi *et al.*, 2015 ▸) have been discussed in review reports. The known potential of benzo­furan derivatives has motivated us to synthesise some new compounds incorporating this core structure and we herein report the synthesis and crystal structures of 2-(1-benzo­furan-2-yl)-2-oxoethyl 2-nitro­benzoate (I) and 2-(1-benzo­furan-2-yl)-2-oxoethyl 2-amino­benzoate (II).
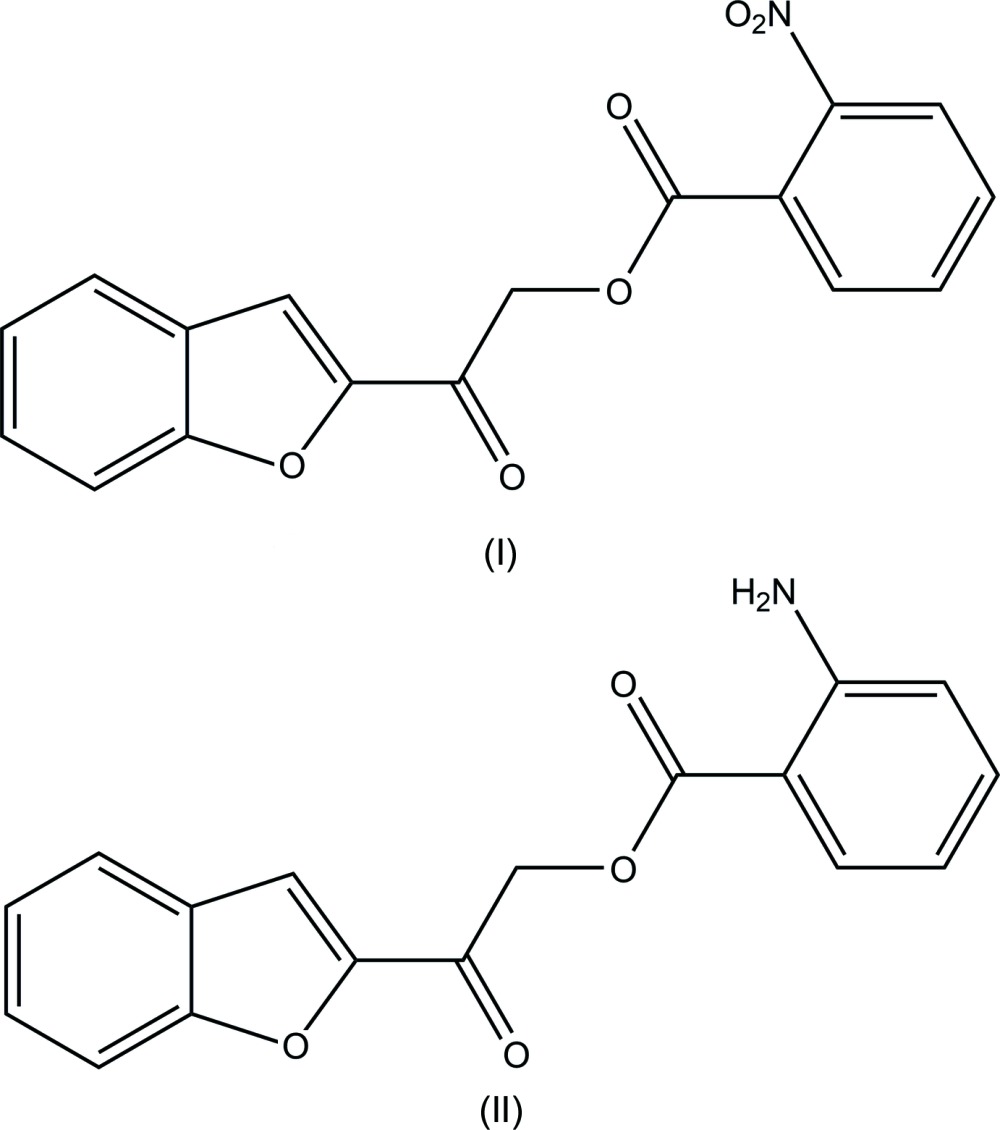


## Structural commentary   

**Figure 1:**
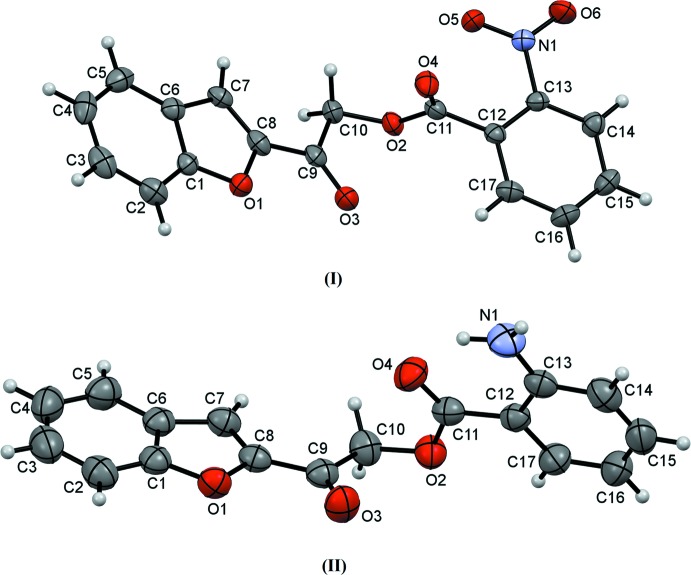
*ORTEP* diagram of the title compounds, with ellipsoids drawn at the 50% probability level, showing the atomic labelling scheme.

**Figure 2:**
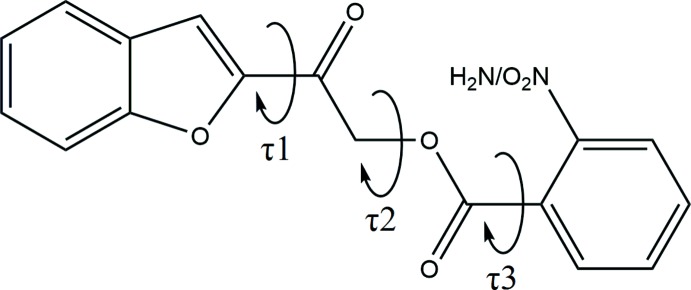
General chemical diagram showing torsion angles τ1, τ2 and τ3 in compounds (I) and (II).

The mol­ecular structures of the title compounds (Fig. 1 ▸) contain a benzo­furan ring and an *ortho*-substituted [nitro- for compound (I) and amino- for compound (II)] phenyl ring, joined by a C—C(=O)—O—C(=O) carbonyl-connecting bridge. Their mol­ecular conformations can be characterized by three degrees of freedom, as indicated by the O1—C8—C9—O3 (τ1), C9—C10—O2—C11 (τ2) and O4—C11—C12—C13 (τ3) torsion angles, respectively (Fig. 2 ▸). The torsion angle τ1 for compounds (I) and (II) is close to 0°, showing that the benzo­furan ring is nearly coplanar with the C—C(=O)—O—C(=O) carbonyl bridge. Torsion angle τ2 adopts a *syn*-clinal conformation, as both carbonyl groups at the connecting bridges are twisted away from each other forming torsion angles of −71.43 (3)° in (I) and −70.85 (18)° in (II). For compound (I), the substituted *ortho*-nitro­phenyl moiety is perpendicular to the adjacent carbonyl group with a τ3 torsion angle of −90.2 (4)°; this may arise from a steric repulsion force between the nitro group and carbonyl group. In contrast, the *ortho*-amino­phenyl ring in compound (II) is almost coplanar with its adjacent carbonyl group due to the intra­molecular hydrogen bond (N1—H1*A*⋯O4, Table 2 ▸) between the amino and carbonyl groups, which generates an *S*(6) ring.

## Supra­molecular features   

**Figure 3:**
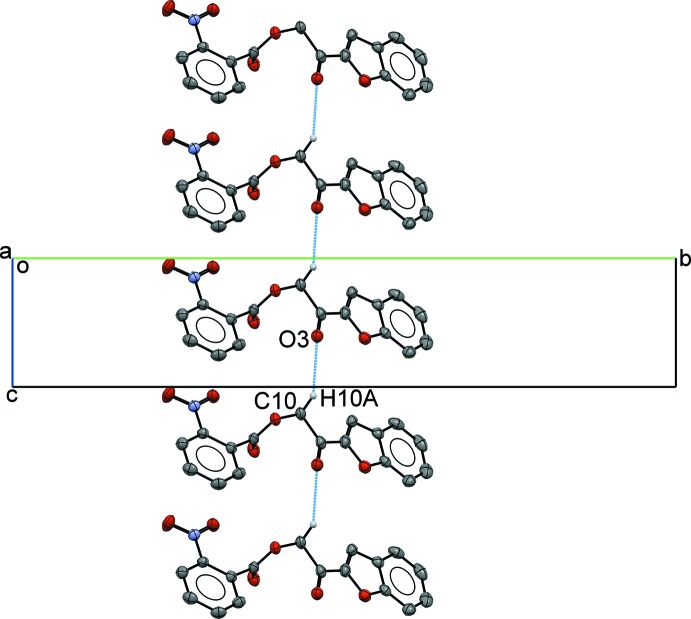
Mol­ecules in compound (I) joined by inter­molecular hydrogen bonds, forming a fishbone chain.

**Figure 4:**
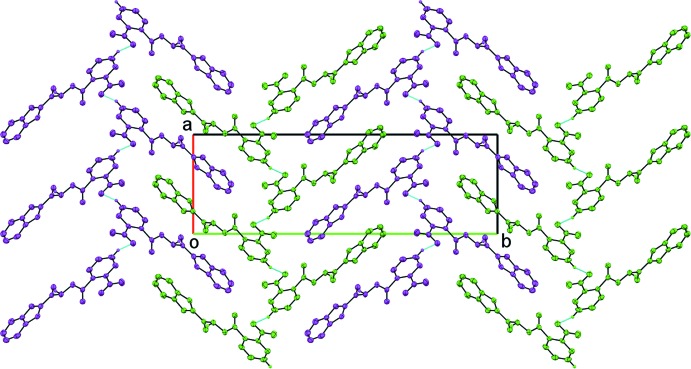
Fishbone chains in an up–down manner are shown in different colours.

The crystal packing of compound (I) depends mainly on two weak inter­molecular hydrogen bonds. Mol­ecules are joined into infinite chains propagating along the *c*-axis by C10—H10*A*⋯O3 hydrogen bonds (Table 1 ▸, Fig. 3 ▸), meanwhile those chains are inter­digitated into a fishbone sheet extending along the [20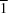] direction through C15—H15*A*⋯O5 hydrogen bonds. The fishbone sheets alternate in an up–down manner along the *ab* plane as shown in Fig. 4 ▸.

**Figure 5:**
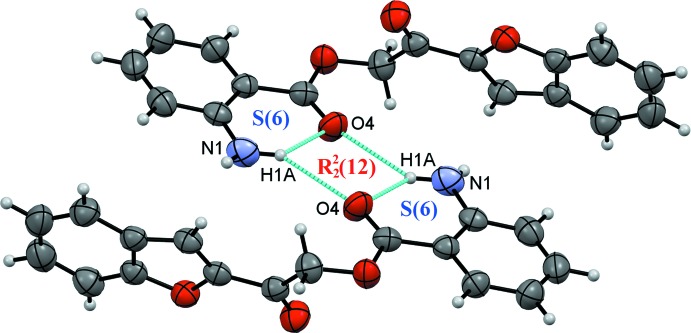
Intra­molecular and inter­molecular N1—H1*A*⋯O4 hydrogen bonds.

**Figure 6:**
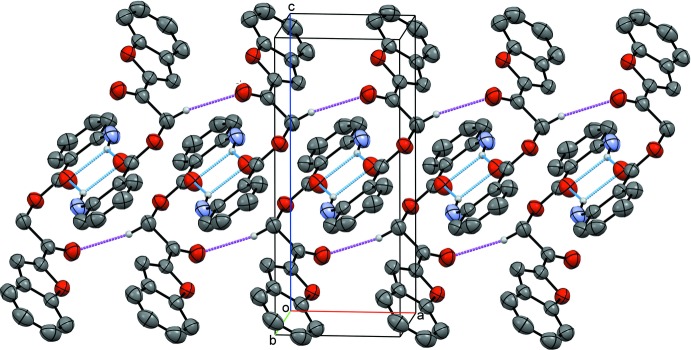
Inter­actions in the crystal structure of compound (II), showing hydrogen bonds (cyan dotted lines) and π⋯π inter­actions (red dotted lines).

**Figure 7:**
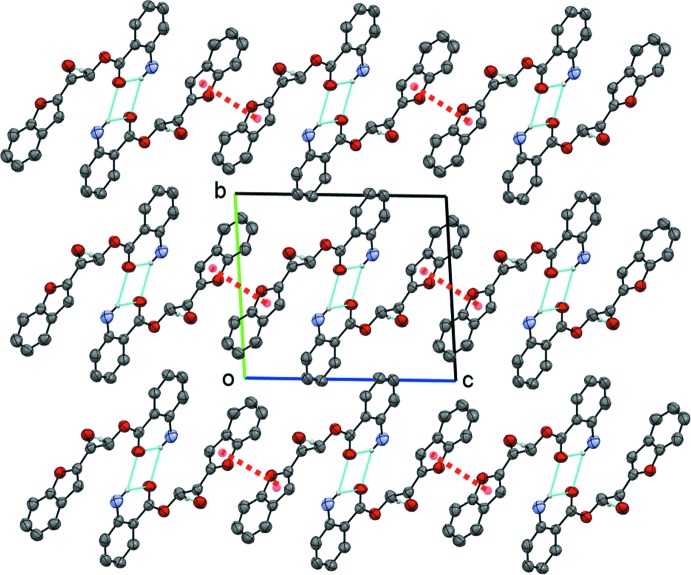
The packing of compound (II), showing the hydrogen bonds (cyan dotted lines) and π–π inter­actions (red dotted lines).

In compound (II), the mol­ecular inter­actions are more abundant than in (I) because of the *ortho*-substituted amino group at its phenyl ring. Pairs of N1—H1*A*⋯O4 hydrogen bonds link mol­ecules into inversion dimers with an 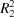
(12) graph-set motif (Fig. 5 ▸). These dimers are further expanded by C10—H10*A*⋯O3 hydrogen bonds into infinite chains along the [100] direction (Fig. 6 ▸). In addition, neighbouring chains are inter­connected by π–π inter­actions involving adjacent furan rings [centroid–centroid distance = 3.7982 (15) Å; symmetry code: −*x*, −*y* + 1, −*z*), forming a sheet parallel to the *ac* plane (Fig. 7 ▸).

## Database survey   

A survey of the Cambridge Structural Database (Groom *et al.*, 2016 ▸) revealed five benzo­furan structures (Kumar *et al.*, 2015 ▸) similar to the title compounds: ITAXUY, ITAYAF, ITAYEJ, ITAYIN and ITAYOT. The mol­ecular structures of the studied and previous compounds differ only at their substituted phenyl rings. By comparing their torsion angles at the C(=O)—O—C(=O) carbonyl bridges, the title compounds exhibit a *syn*-clinal conformation similar to ITAXUY, ITAYEJ and ITAYIN with respect to their torsion angles which range from 75 to 80°.

## Synthesis and crystallization   

The synthesis was carried out by reacting 1-(benzo­furan-2-yl)-2-bromo­ethan-1-one (1 mmol) with 2-nitro­benzoic acid (1 mmol) for compound (I) and 2-amino­benzoic acid (1 mmol) for compound (II) in 8 ml of *N*,*N*-di­methyl­formamide in the presence of a catalytic amount of anhydrous potassium carbonate at room temperature. The reaction solution was stirred for about two h and monitored by thin-layer chromatography (TLC). After the reaction was complete, the resultant mixture was then added to a beaker of ice-cooled water to form a precipitate. The precipitate was then filtered, rinsed with distilled water and dried. Crystals suitable for X-ray analysis were obtained by slow evaporation using a suitable solvent.

**2-(Benzo­furan-2-yl)-2-oxoethyl 2-nitro­benzoate (I):**

Solvents used to grow crystal: acetone + methanol 1:1 *v*/*v*); yield: 80%, m.p. 381–383 K; ^1^H NMR (500MHz, CDCl_3_) in ppm: *δ* 8.041–8.025 (*d*, 1H, *J =* 7.9Hz, ^14^CH), 7.995–7.980 (*d*, 1H, *J =* 7.9Hz, ^17^CH), 7.796–7.763 (*m*, 2H, ^2^CH, ^3^CH), 7.726–7.695 (*t*, 1H, *J =* 7.9Hz, ^15^CH), 7.673 (*s*, 1H, ^7^CH), 7.644–7.627 (*d*, 1H, *J =* 8.4Hz, ^5^CH), 7.578–7.544 (*t*, 1H, *J =* 8.4Hz, ^4^CH), 7.398–7.366 (*t*, 1H, *J =* 7.9Hz, ^16^CH), 5.609 (*s*, 2H, ^10^CH_2_). ^13^C NMR (125 MHz, CDCl_3_) in ppm: 182.94 (C9), 165.67 (C11), 155.80 (C1), 150.25 (C13), 133.31 (C16), 132.04 (C15), 130.39 (C17), 130.10 (C8), 128.97 (C3), 127.23 (C12), 126.70 (C6), 124.34 (C5), 124.11 (C4), 123.60 (C14), 113.75 (C7), 112.57 (C2), 67.10 (C10). FT–IR (ATR (solid) cm^−1^): 3089 (Ar C—H, ν), 2953 (C—H, ν), 1744, 1686 (C=O, ν), 1612 (C=C, ν), 1554, 1422 (Ar C=C, ν), 1529, 1344 (N=O, ν), 1278, 1123 (C—O, ν).

**2-(Benzo­furan-2-yl)-2-oxoethyl 2-amino­benzoate (II)**:

Solvents used to grow crystal: acetone + aceto­nitrile (1:1 *v*/*v*); yield: 83%; m.p. 432–434 K; ^1^H NMR (500 MHz, DMSO) in ppm: *δ* 8.083 (*s*, 1H, ^7^CH), 7.907–7.891 (*d*, 1H, *J* = 8.1Hz, ^17^CH), 7.848–7.832 (*d*, 1H, *J =* 8.1Hz, ^14^CH), 7.787–7.770 (*d*, 1H, *J =* 8.5Hz, ^2^CH), 7.617–7.583 (*t*, 1H, *J =* 8.5Hz, ^3^CH), 7.437–7.405 (*t*, 1H, *J =* 8.1Hz, ^15^CH), 7.329–7.295 (*t*, 1H, *J =* 8.5Hz, ^4^CH), 6.824–6.807 (*d*, 1H, *J =* 8.5Hz, ^5^CH), 6.669 (*br*–*s*, 2H, ^1^NH_2_), 6.607–6.574 (*t*, 1H, *J =* 8.1Hz, ^16^CH), 5.591 (*s*, 2H, ^10^CH_2_). ^13^C NMR (125MHz, DMSO) in ppm: 184.08 (C9), 166.60 (C11), 154.96 (C1), 151.62 (C15), 149.63 (C13), 134.49 (C8), 130.78 (C17), 128.88 (C3), 126.49 (C6), 124.28 (C5), 123.84 (C4), 116.63 (C14), 114.84 (C16), 114.66 (C7), 112.31 (C2), 107.87 (C2), 65.63 (C10). FT–IR (ATR (solid) cm^−1^): 3473, 3360 (N—H, ν), 3078 (Ar C—H, ν), 2942 (C—H, ν), 1697, 1676 (C=O, ν), 1615 (C=C, ν), 1583, 1487 (Ar C=C, ν), 1244, 1112 (C—O, ν).

## Refinement   

Crystal data, data collection and structure refinement details for both compounds are summarized in Table 3 ▸. All C-bound H atoms were positioned geometrically (C—H = 0.93–0.97 Å) and refined using a riding model with *U*_iso_(H) = 1.2*U*_eq_(parent atom). The N-bound H atoms of compound (II) were located in a difference-Fourier map and refined freely.

## Supplementary Material

Crystal structure: contains datablock(s) I, II. DOI: 10.1107/S2056989017009422/qm2116sup1.cif

Structure factors: contains datablock(s) mo_bzf12_0m. DOI: 10.1107/S2056989017009422/qm2116Isup2.hkl

Supporting information file. DOI: 10.1107/S2056989017009422/qm2116Isup4.cml

Structure factors: contains datablock(s) II. DOI: 10.1107/S2056989017009422/qm2116IIsup3.hkl

Supporting information file. DOI: 10.1107/S2056989017009422/qm2116IIsup5.cml

CCDC references: 1449589, 1449587

Additional supporting information:  crystallographic information; 3D view; checkCIF report

## Figures and Tables

**Table 1:** Hydrogen-bond geometry (Å, °) for (I)

*D*—H⋯*A*	*D*—H	H⋯*A*	*D*⋯*A*	*D*—H⋯*A*
C10—H10*A*⋯O3^i^	0.99	2.59	3.471 (4)	148
C15—H15*A*⋯O5^ii^	0.95	2.58	3.380 (3)	142

Symmetry codes: (i) 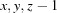; (ii) 
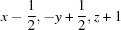
.

**Table 2:** Hydrogen-bond geometry (Å, °) for (II)

*D*—H⋯*A*	*D*—H	H⋯*A*	*D*⋯*A*	*D*—H⋯*A*
N1—H1*A*⋯O4	0.91 (2)	2.05 (2)	2.700 (3)	127.7 (18)
N1—H1*A*⋯O4^i^	0.91 (2)	2.49 (2)	3.246 (2)	141.4 (18)
C10—H10*A*⋯O3^ii^	0.97	2.50	3.444 (2)	165

Symmetry codes: (i) 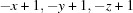; (ii) 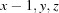.

**Table 3:** Experimental details

	(I)	(II)
Crystal data		
Chemical formula	C_17_H_11_NO_6_	C_17_H_13_NO_4_
*M*_r_	325.27	295.28
Crystal system, space group	Orthorhombic, *P* *n* *a*2_1_	Triclinic, *P* 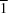
Temperature (K)	100	297
*a*, *b*, *c* (Å)	9.3022 (10), 28.482 (3), 5.5208 (6)	5.1839 (12), 10.853 (3), 12.269 (3)
α, β, γ (°)	90, 90, 90	93.562 (3), 91.167 (3), 98.714 (3)
*V* (Å^3^)	1462.7 (3)	680.6 (3)
*Z*	4	2
Radiation type	Mo *K*α	Mo *K*α
μ (mm^−1^)	0.11	0.10
Crystal size (mm)	0.27 × 0.16 × 0.13	0.40 × 0.32 × 0.21

Data collection		
Diffractometer	Bruker APEXII DUO CCD area-detector	Bruker APEXII DUO CCD area-detector
Absorption correction	Multi-scan (*SADABS*; Bruker, 2009 ▸)	Multi-scan (*SADABS*; Bruker, 2009 ▸)
*T*_min_, *T*_max_	0.933, 0.985	0.871, 0.978
No. of measured, independent and observed [*I* > 2σ(*I*)] reflections	15875, 3358, 2915	17052, 3105, 2214
*R*_int_	0.037	0.037
(sin θ/λ)_max_ (Å^−1^)	0.651	0.650

Refinement		
*R*[*F* ^2^ > 2σ(*F* ^2^)], *wR*(*F* ^2^), *S*	0.038, 0.085, 1.08	0.046, 0.123, 1.08
No. of reflections	3358	3105
No. of parameters	217	207
No. of restraints	1	0
H-atom treatment	H-atom parameters constrained	H atoms treated by a mixture of independent and constrained refinement
Δρ_max_, Δρ_min_ (e Å^−3^)	0.18, −0.17	0.18, −0.18

Computer programs: *APEX2* and *SAINT* (Bruker, 2009 ▸), *SHELXT2013* (Sheldrick, 2015*a* ▸), *SHELXL2013* (Sheldrick, 2015*b* ▸), *Mercury* (Macrae *et al.*, 2006 ▸) and *PLATON* (Spek, 2009 ▸).

## supplementary crystallographic information

### (I) 2-(1H-1-Benzofuran-2-yl)-2-oxoethyl 2-nitrobenzoate Crystal data

**Table:**

C_17_H_11_NO_6_	*D*_x_ = 1.477 Mg m^−^^3^
*M**_r_* = 325.27	Mo *K*α radiation, λ = 0.71073 Å
Orthorhombic, *P**n**a*2_1_	Cell parameters from 3687 reflections
*a* = 9.3022 (10) Å	θ = 2.3–25.3°
*b* = 28.482 (3) Å	µ = 0.11 mm^−^^1^
*c* = 5.5208 (6) Å	*T* = 100 K
*V* = 1462.7 (3) Å^3^	Block, colourless
*Z* = 4	0.27 × 0.16 × 0.13 mm
*F*(000) = 672	

### (I) 2-(1H-1-Benzofuran-2-yl)-2-oxoethyl 2-nitrobenzoate Data collection

**Table:**

Bruker APEXII DUO CCD area-detector diffractometer	3358 independent reflections
Radiation source: fine-focus sealed tube	2915 reflections with *I* > 2σ(*I*)
Graphite monochromator	*R*_int_ = 0.037
φ and ω scans	θ_max_ = 27.6°, θ_min_ = 1.4°
Absorption correction: multi-scan (SADABS; Bruker, 2009)	*h* = −12→12
*T*_min_ = 0.933, *T*_max_ = 0.985	*k* = −36→37
15875 measured reflections	*l* = −7→7

### (I) 2-(1H-1-Benzofuran-2-yl)-2-oxoethyl 2-nitrobenzoate Refinement

**Table:**

Refinement on *F*^2^	1 restraint
Least-squares matrix: full	Hydrogen site location: inferred from neighbouring sites
*R*[*F*^2^ > 2σ(*F*^2^)] = 0.038	H-atom parameters constrained
*wR*(*F*^2^) = 0.085	*w* = 1/[σ^2^(*F*_o_^2^) + (0.0345*P*)^2^ + 0.279*P*], where *P* = (*F*_o_^2^ + 2*F*_c_^2^)/3
*S* = 1.08	(Δ/σ)_max_ < 0.001
3358 reflections	Δρ_max_ = 0.18 e Å^−^^3^
217 parameters	Δρ_min_ = −0.17 e Å^−^^3^

### (I) 2-(1H-1-Benzofuran-2-yl)-2-oxoethyl 2-nitrobenzoate Special details

**Table:**

Geometry. All esds (except the esd in the dihedral angle between two l.s. planes) are estimated using the full covariance matrix. The cell esds are taken into account individually in the estimation of esds in distances, angles and torsion angles; correlations between esds in cell parameters are only used when they are defined by crystal symmetry. An approximate (isotropic) treatment of cell esds is used for estimating esds involving l.s. planes.

### (I) 2-(1H-1-Benzofuran-2-yl)-2-oxoethyl 2-nitrobenzoate Fractional atomic coordinates and isotropic or equivalent isotropic displacement parameters (Å^2^)

**Table:**

	*x*	*y*	*z*	*U*_iso_*/*U*_eq_	
N1	0.5075 (2)	0.27391 (8)	0.1493 (4)	0.0290 (5)	
O1	0.72961 (19)	0.53112 (6)	0.6220 (3)	0.0309 (4)	
O2	0.51143 (18)	0.39614 (6)	0.2506 (3)	0.0289 (4)	
O3	0.5376 (2)	0.45933 (6)	0.6044 (4)	0.0328 (4)	
O4	0.6811 (2)	0.36342 (6)	0.4887 (4)	0.0350 (5)	
O5	0.5946 (2)	0.30243 (6)	0.0721 (4)	0.0343 (4)	
O6	0.4877 (2)	0.23524 (7)	0.0592 (4)	0.0460 (6)	
C1	0.8415 (3)	0.56187 (8)	0.5803 (5)	0.0270 (6)	
C2	0.8830 (3)	0.59794 (10)	0.7294 (6)	0.0362 (6)	
H2A	0.8343	0.6045	0.8768	0.043*	
C3	0.9982 (3)	0.62384 (10)	0.6543 (6)	0.0412 (7)	
H3A	1.0313	0.6488	0.7539	0.049*	
C4	1.0686 (3)	0.61491 (10)	0.4372 (6)	0.0413 (8)	
H4A	1.1475	0.6341	0.3910	0.050*	
C5	1.0259 (3)	0.57859 (11)	0.2866 (6)	0.0380 (7)	
H5A	1.0743	0.5725	0.1385	0.046*	
C6	0.9074 (3)	0.55093 (9)	0.3613 (5)	0.0278 (6)	
C7	0.8303 (3)	0.51167 (9)	0.2669 (5)	0.0284 (6)	
H7A	0.8489	0.4958	0.1187	0.034*	
C8	0.7272 (3)	0.50142 (9)	0.4259 (5)	0.0312 (6)	
C9	0.6161 (3)	0.46488 (9)	0.4317 (5)	0.0286 (6)	
C10	0.6080 (3)	0.43440 (9)	0.2082 (5)	0.0299 (6)	
H10A	0.5737	0.4533	0.0691	0.036*	
H10B	0.7047	0.4221	0.1685	0.036*	
C11	0.5621 (3)	0.36359 (9)	0.4045 (5)	0.0272 (6)	
C12	0.4463 (3)	0.32984 (8)	0.4742 (5)	0.0250 (5)	
C13	0.4219 (3)	0.28707 (9)	0.3614 (4)	0.0247 (5)	
C14	0.3186 (3)	0.25567 (9)	0.4415 (5)	0.0307 (6)	
H14A	0.3052	0.2264	0.3622	0.037*	
C15	0.2354 (3)	0.26790 (10)	0.6397 (5)	0.0345 (6)	
H15A	0.1640	0.2469	0.6979	0.041*	
C16	0.2562 (3)	0.31066 (10)	0.7531 (5)	0.0348 (6)	
H16A	0.1978	0.3191	0.8874	0.042*	
C17	0.3614 (3)	0.34129 (10)	0.6725 (5)	0.0319 (6)	
H17A	0.3755	0.3704	0.7535	0.038*	

### (I) 2-(1H-1-Benzofuran-2-yl)-2-oxoethyl 2-nitrobenzoate Atomic displacement parameters (Å^2^)

**Table:**

	*U*^11^	*U*^22^	*U*^33^	*U*^12^	*U*^13^	*U*^23^
N1	0.0311 (11)	0.0268 (11)	0.0292 (12)	0.0017 (9)	0.0055 (10)	−0.0006 (10)
O1	0.0304 (10)	0.0307 (9)	0.0316 (9)	0.0006 (8)	0.0030 (8)	0.0025 (8)
O2	0.0337 (10)	0.0216 (9)	0.0314 (9)	0.0024 (7)	−0.0008 (9)	0.0025 (8)
O3	0.0371 (10)	0.0267 (9)	0.0346 (10)	0.0017 (8)	0.0038 (9)	−0.0001 (8)
O4	0.0278 (10)	0.0305 (10)	0.0467 (12)	0.0005 (8)	−0.0025 (9)	0.0064 (9)
O5	0.0374 (10)	0.0291 (9)	0.0364 (11)	0.0010 (8)	0.0133 (9)	0.0021 (9)
O6	0.0516 (13)	0.0338 (11)	0.0527 (13)	−0.0047 (10)	0.0184 (11)	−0.0161 (10)
C1	0.0267 (13)	0.0262 (12)	0.0280 (13)	0.0043 (10)	−0.0017 (11)	0.0067 (11)
C2	0.0419 (16)	0.0360 (16)	0.0307 (14)	0.0065 (13)	−0.0075 (13)	−0.0020 (12)
C3	0.0426 (17)	0.0341 (15)	0.0470 (18)	0.0033 (13)	−0.0196 (15)	0.0017 (14)
C4	0.0293 (15)	0.0395 (17)	0.055 (2)	−0.0040 (13)	−0.0109 (15)	0.0193 (15)
C5	0.0326 (15)	0.0502 (18)	0.0312 (15)	0.0123 (13)	0.0035 (13)	0.0142 (13)
C6	0.0281 (13)	0.0299 (14)	0.0256 (13)	0.0072 (11)	−0.0027 (11)	0.0043 (11)
C7	0.0316 (14)	0.0247 (13)	0.0289 (13)	0.0081 (11)	−0.0035 (12)	−0.0022 (11)
C8	0.0349 (14)	0.0230 (12)	0.0357 (15)	0.0058 (11)	−0.0062 (13)	−0.0002 (11)
C9	0.0293 (13)	0.0222 (12)	0.0342 (14)	0.0065 (11)	−0.0007 (12)	0.0042 (11)
C10	0.0343 (14)	0.0208 (12)	0.0345 (15)	0.0017 (11)	0.0044 (12)	0.0032 (11)
C11	0.0299 (14)	0.0217 (12)	0.0299 (14)	0.0059 (11)	0.0028 (12)	−0.0010 (11)
C12	0.0234 (12)	0.0244 (12)	0.0273 (13)	0.0056 (10)	0.0004 (11)	0.0021 (11)
C13	0.0233 (12)	0.0281 (13)	0.0227 (12)	0.0052 (10)	0.0021 (10)	0.0008 (11)
C14	0.0301 (13)	0.0308 (14)	0.0313 (13)	−0.0033 (11)	−0.0001 (12)	−0.0007 (12)
C15	0.0275 (13)	0.0436 (16)	0.0324 (14)	−0.0054 (12)	0.0042 (12)	0.0049 (13)
C16	0.0289 (14)	0.0471 (17)	0.0286 (13)	0.0011 (13)	0.0069 (12)	−0.0014 (14)
C17	0.0320 (14)	0.0343 (15)	0.0295 (14)	0.0050 (12)	0.0012 (12)	−0.0051 (12)

### (I) 2-(1H-1-Benzofuran-2-yl)-2-oxoethyl 2-nitrobenzoate Geometric parameters (Å, º)

**Table:**

N1—O6	1.223 (3)	C6—C7	1.427 (4)
N1—O5	1.224 (3)	C7—C8	1.333 (4)
N1—C13	1.465 (3)	C7—H7A	0.9500
O1—C8	1.374 (3)	C8—C9	1.467 (4)
O1—C1	1.380 (3)	C9—C10	1.511 (4)
O2—C11	1.343 (3)	C10—H10A	0.9900
O2—C10	1.431 (3)	C10—H10B	0.9900
O3—C9	1.211 (3)	C11—C12	1.494 (4)
O4—C11	1.201 (3)	C12—C13	1.387 (3)
C1—C2	1.372 (4)	C12—C17	1.389 (4)
C1—C6	1.391 (4)	C13—C14	1.385 (4)
C2—C3	1.365 (4)	C14—C15	1.385 (4)
C2—H2A	0.9500	C14—H14A	0.9500
C3—C4	1.389 (5)	C15—C16	1.383 (4)
C3—H3A	0.9500	C15—H15A	0.9500
C4—C5	1.385 (4)	C16—C17	1.384 (4)
C4—H4A	0.9500	C16—H16A	0.9500
C5—C6	1.416 (4)	C17—H17A	0.9500
C5—H5A	0.9500		
			
O6—N1—O5	123.8 (2)	O3—C9—C10	122.6 (2)
O6—N1—C13	118.3 (2)	C8—C9—C10	115.1 (2)
O5—N1—C13	117.9 (2)	O2—C10—C9	109.6 (2)
C8—O1—C1	105.8 (2)	O2—C10—H10A	109.8
C11—O2—C10	114.1 (2)	C9—C10—H10A	109.8
C2—C1—O1	126.0 (3)	O2—C10—H10B	109.8
C2—C1—C6	124.4 (3)	C9—C10—H10B	109.8
O1—C1—C6	109.6 (2)	H10A—C10—H10B	108.2
C3—C2—C1	116.3 (3)	O4—C11—O2	124.9 (2)
C3—C2—H2A	121.8	O4—C11—C12	124.2 (2)
C1—C2—H2A	121.8	O2—C11—C12	110.7 (2)
C2—C3—C4	122.2 (3)	C13—C12—C17	117.8 (2)
C2—C3—H3A	118.9	C13—C12—C11	124.6 (2)
C4—C3—H3A	118.9	C17—C12—C11	117.5 (2)
C5—C4—C3	121.3 (3)	C14—C13—C12	122.5 (2)
C5—C4—H4A	119.4	C14—C13—N1	117.8 (2)
C3—C4—H4A	119.4	C12—C13—N1	119.7 (2)
C4—C5—C6	117.6 (3)	C13—C14—C15	118.5 (3)
C4—C5—H5A	121.2	C13—C14—H14A	120.7
C6—C5—H5A	121.2	C15—C14—H14A	120.7
C1—C6—C5	118.1 (3)	C16—C15—C14	120.1 (3)
C1—C6—C7	105.7 (2)	C16—C15—H15A	120.0
C5—C6—C7	136.1 (3)	C14—C15—H15A	120.0
C8—C7—C6	107.0 (2)	C15—C16—C17	120.6 (3)
C8—C7—H7A	126.5	C15—C16—H16A	119.7
C6—C7—H7A	126.5	C17—C16—H16A	119.7
C7—C8—O1	111.9 (2)	C16—C17—C12	120.5 (3)
C7—C8—C9	132.6 (3)	C16—C17—H17A	119.8
O1—C8—C9	115.5 (2)	C12—C17—H17A	119.8
O3—C9—C8	122.3 (3)		
			
C8—O1—C1—C2	179.6 (2)	O3—C9—C10—O2	−7.9 (3)
C8—O1—C1—C6	−0.3 (3)	C8—C9—C10—O2	171.4 (2)
O1—C1—C2—C3	179.2 (2)	C10—O2—C11—O4	−5.4 (4)
C6—C1—C2—C3	−0.8 (4)	C10—O2—C11—C12	169.6 (2)
C1—C2—C3—C4	1.1 (4)	O4—C11—C12—C13	−90.2 (4)
C2—C3—C4—C5	−0.8 (4)	O2—C11—C12—C13	94.7 (3)
C3—C4—C5—C6	0.3 (4)	O4—C11—C12—C17	86.9 (3)
C2—C1—C6—C5	0.3 (4)	O2—C11—C12—C17	−88.2 (3)
O1—C1—C6—C5	−179.7 (2)	C17—C12—C13—C14	−1.0 (4)
C2—C1—C6—C7	−179.7 (2)	C11—C12—C13—C14	176.1 (2)
O1—C1—C6—C7	0.3 (3)	C17—C12—C13—N1	179.1 (2)
C4—C5—C6—C1	0.0 (4)	C11—C12—C13—N1	−3.8 (4)
C4—C5—C6—C7	−180.0 (3)	O6—N1—C13—C14	−2.8 (3)
C1—C6—C7—C8	−0.1 (3)	O5—N1—C13—C14	177.0 (2)
C5—C6—C7—C8	179.9 (3)	O6—N1—C13—C12	177.1 (2)
C6—C7—C8—O1	−0.1 (3)	O5—N1—C13—C12	−3.1 (3)
C6—C7—C8—C9	−179.1 (3)	C12—C13—C14—C15	1.0 (4)
C1—O1—C8—C7	0.3 (3)	N1—C13—C14—C15	−179.1 (2)
C1—O1—C8—C9	179.4 (2)	C13—C14—C15—C16	0.0 (4)
C7—C8—C9—O3	174.5 (3)	C14—C15—C16—C17	−1.0 (4)
O1—C8—C9—O3	−4.5 (3)	C15—C16—C17—C12	1.0 (4)
C7—C8—C9—C10	−4.9 (4)	C13—C12—C17—C16	0.1 (4)
O1—C8—C9—C10	176.2 (2)	C11—C12—C17—C16	−177.3 (2)
C11—O2—C10—C9	−71.4 (3)		

### (I) 2-(1H-1-Benzofuran-2-yl)-2-oxoethyl 2-nitrobenzoate Hydrogen-bond geometry (Å, º)

**Table:**

*D*—H···*A*	*D*—H	H···*A*	*D*···*A*	*D*—H···*A*
C10—H10*A*···O3^i^	0.99	2.59	3.471 (4)	148
C15—H15*A*···O5^ii^	0.95	2.58	3.380 (3)	142

Symmetry codes: (i) *x*, *y*, *z*−1; (ii) *x*−1/2, −*y*+1/2, *z*+1.

### (II) 2-(1H-1-Benzofuran-2-yl)-2-oxoethyl 2-aminobenzoate Crystal data

**Table:**

C_17_H_13_NO_4_	*Z* = 2
*M**_r_* = 295.28	*F*(000) = 308
Triclinic, *P*1	*D*_x_ = 1.441 Mg m^−^^3^
*a* = 5.1839 (12) Å	Mo *K*α radiation, λ = 0.71073 Å
*b* = 10.853 (3) Å	Cell parameters from 5796 reflections
*c* = 12.269 (3) Å	θ = 2.4–27.9°
α = 93.562 (3)°	µ = 0.10 mm^−^^1^
β = 91.167 (3)°	*T* = 297 K
γ = 98.714 (3)°	Block, orange
*V* = 680.6 (3) Å^3^	0.40 × 0.32 × 0.21 mm

### (II) 2-(1H-1-Benzofuran-2-yl)-2-oxoethyl 2-aminobenzoate Data collection

**Table:**

Bruker APEXII DUO CCD area-detector diffractometer	3105 independent reflections
Radiation source: fine-focus sealed tube	2214 reflections with *I* > 2σ(*I*)
Graphite monochromator	*R*_int_ = 0.037
φ and ω scans	θ_max_ = 27.5°, θ_min_ = 1.7°
Absorption correction: multi-scan (SADABS; Bruker, 2009)	*h* = −6→6
*T*_min_ = 0.871, *T*_max_ = 0.978	*k* = −14→14
17052 measured reflections	*l* = −15→15

### (II) 2-(1H-1-Benzofuran-2-yl)-2-oxoethyl 2-aminobenzoate Refinement

**Table:**

Refinement on *F*^2^	0 restraints
Least-squares matrix: full	Hydrogen site location: mixed
*R*[*F*^2^ > 2σ(*F*^2^)] = 0.046	H atoms treated by a mixture of independent and constrained refinement
*wR*(*F*^2^) = 0.123	*w* = 1/[σ^2^(*F*_o_^2^) + (0.0462*P*)^2^ + 0.1899*P*] where *P* = (*F*_o_^2^ + 2*F*_c_^2^)/3
*S* = 1.08	(Δ/σ)_max_ < 0.001
3105 reflections	Δρ_max_ = 0.18 e Å^−^^3^
207 parameters	Δρ_min_ = −0.18 e Å^−^^3^

### (II) 2-(1H-1-Benzofuran-2-yl)-2-oxoethyl 2-aminobenzoate Special details

**Table:**

Geometry. All esds (except the esd in the dihedral angle between two l.s. planes) are estimated using the full covariance matrix. The cell esds are taken into account individually in the estimation of esds in distances, angles and torsion angles; correlations between esds in cell parameters are only used when they are defined by crystal symmetry. An approximate (isotropic) treatment of cell esds is used for estimating esds involving l.s. planes.

### (II) 2-(1H-1-Benzofuran-2-yl)-2-oxoethyl 2-aminobenzoate Fractional atomic coordinates and isotropic or equivalent isotropic displacement parameters (Å^2^)

**Table:**

	*x*	*y*	*z*	*U*_iso_*/*U*_eq_	
N1	0.6563 (4)	0.67887 (18)	0.63733 (16)	0.0583 (4)	
H1A	0.611 (4)	0.617 (2)	0.5840 (19)	0.069 (7)*	
H1B	0.797 (5)	0.682 (2)	0.6702 (19)	0.074 (7)*	
O1	0.2314 (2)	0.47254 (11)	0.10178 (9)	0.0478 (3)	
O2	0.0514 (2)	0.72867 (11)	0.41702 (9)	0.0500 (3)	
O3	0.3365 (3)	0.67869 (12)	0.24247 (11)	0.0587 (4)	
O4	0.2949 (3)	0.59366 (11)	0.47797 (11)	0.0579 (4)	
C1	0.1297 (3)	0.35882 (16)	0.05120 (14)	0.0448 (4)	
C2	0.2360 (4)	0.3001 (2)	−0.03521 (16)	0.0581 (5)	
H2A	0.3890	0.3356	−0.0667	0.070*	
C3	0.1032 (5)	0.1866 (2)	−0.07204 (17)	0.0677 (6)	
H3A	0.1669	0.1434	−0.1309	0.081*	
C4	−0.1240 (5)	0.1334 (2)	−0.02449 (18)	0.0672 (6)	
H4A	−0.2072	0.0551	−0.0514	0.081*	
C5	−0.2283 (4)	0.19380 (18)	0.06135 (17)	0.0585 (5)	
H5A	−0.3814	0.1581	0.0926	0.070*	
C6	−0.0976 (3)	0.31045 (16)	0.10033 (14)	0.0452 (4)	
C7	−0.1376 (3)	0.40069 (16)	0.18454 (14)	0.0453 (4)	
H7A	−0.2762	0.3957	0.2318	0.054*	
C8	0.0625 (3)	0.49452 (16)	0.18298 (13)	0.0429 (4)	
C9	0.1355 (3)	0.60838 (16)	0.25240 (14)	0.0439 (4)	
C10	−0.0582 (3)	0.63287 (18)	0.33799 (14)	0.0507 (4)	
H10A	−0.2107	0.6571	0.3031	0.061*	
H10B	−0.1141	0.5567	0.3741	0.061*	
C11	0.2338 (3)	0.69697 (16)	0.48521 (13)	0.0431 (4)	
C12	0.3415 (3)	0.79867 (15)	0.56403 (13)	0.0407 (4)	
C13	0.5539 (3)	0.78708 (16)	0.63365 (13)	0.0441 (4)	
C14	0.6573 (4)	0.89064 (19)	0.70265 (15)	0.0567 (5)	
H14A	0.7996	0.8855	0.7486	0.068*	
C15	0.5549 (4)	0.9990 (2)	0.70424 (16)	0.0611 (5)	
H15A	0.6289	1.0667	0.7506	0.073*	
C16	0.3431 (4)	1.00947 (18)	0.63798 (16)	0.0603 (5)	
H16A	0.2715	1.0831	0.6403	0.072*	
C17	0.2401 (4)	0.91065 (17)	0.56910 (15)	0.0515 (4)	
H17A	0.0976	0.9179	0.5240	0.062*	

### (II) 2-(1H-1-Benzofuran-2-yl)-2-oxoethyl 2-aminobenzoate Atomic displacement parameters (Å^2^)

**Table:**

	*U*^11^	*U*^22^	*U*^33^	*U*^12^	*U*^13^	*U*^23^
N1	0.0538 (10)	0.0643 (11)	0.0604 (11)	0.0199 (9)	−0.0078 (8)	0.0097 (9)
O1	0.0432 (7)	0.0505 (7)	0.0489 (7)	0.0035 (5)	0.0037 (5)	0.0054 (5)
O2	0.0511 (7)	0.0514 (7)	0.0492 (7)	0.0158 (6)	−0.0066 (6)	−0.0011 (6)
O3	0.0505 (8)	0.0579 (8)	0.0635 (8)	−0.0041 (6)	0.0051 (6)	−0.0002 (6)
O4	0.0647 (8)	0.0426 (7)	0.0680 (8)	0.0158 (6)	−0.0080 (7)	0.0023 (6)
C1	0.0456 (9)	0.0457 (10)	0.0441 (9)	0.0097 (8)	−0.0051 (7)	0.0072 (8)
C2	0.0588 (12)	0.0676 (13)	0.0508 (11)	0.0174 (10)	0.0053 (9)	0.0058 (9)
C3	0.0846 (16)	0.0697 (14)	0.0532 (12)	0.0292 (12)	−0.0042 (11)	−0.0027 (10)
C4	0.0846 (16)	0.0519 (12)	0.0630 (13)	0.0095 (11)	−0.0195 (12)	−0.0043 (10)
C5	0.0565 (11)	0.0540 (11)	0.0622 (12)	−0.0018 (9)	−0.0073 (9)	0.0083 (10)
C6	0.0457 (9)	0.0462 (10)	0.0442 (9)	0.0075 (8)	−0.0060 (7)	0.0081 (8)
C7	0.0399 (9)	0.0523 (10)	0.0438 (9)	0.0047 (8)	0.0017 (7)	0.0087 (8)
C8	0.0408 (9)	0.0489 (10)	0.0409 (9)	0.0105 (7)	−0.0014 (7)	0.0093 (7)
C9	0.0405 (9)	0.0458 (10)	0.0458 (9)	0.0065 (8)	−0.0051 (7)	0.0080 (7)
C10	0.0432 (10)	0.0576 (11)	0.0508 (10)	0.0083 (8)	−0.0033 (8)	−0.0008 (8)
C11	0.0422 (9)	0.0447 (10)	0.0444 (9)	0.0098 (7)	0.0042 (7)	0.0093 (7)
C12	0.0416 (9)	0.0414 (9)	0.0403 (8)	0.0078 (7)	0.0058 (7)	0.0064 (7)
C13	0.0417 (9)	0.0511 (10)	0.0404 (9)	0.0064 (8)	0.0069 (7)	0.0104 (7)
C14	0.0538 (11)	0.0684 (13)	0.0461 (10)	0.0039 (10)	−0.0026 (8)	0.0032 (9)
C15	0.0724 (13)	0.0573 (12)	0.0488 (11)	−0.0023 (10)	0.0052 (9)	−0.0049 (9)
C16	0.0776 (14)	0.0471 (11)	0.0579 (11)	0.0156 (10)	0.0074 (10)	−0.0006 (9)
C17	0.0565 (11)	0.0482 (10)	0.0520 (10)	0.0152 (9)	0.0008 (8)	0.0034 (8)

### (II) 2-(1H-1-Benzofuran-2-yl)-2-oxoethyl 2-aminobenzoate Geometric parameters (Å, º)

**Table:**

N1—C13	1.363 (2)	C6—C7	1.419 (2)
N1—H1A	0.91 (2)	C7—C8	1.340 (2)
N1—H1B	0.82 (2)	C7—H7A	0.9300
O1—C1	1.371 (2)	C8—C9	1.453 (2)
O1—C8	1.373 (2)	C9—C10	1.507 (3)
O2—C11	1.3469 (19)	C10—H10A	0.9700
O2—C10	1.420 (2)	C10—H10B	0.9700
O3—C9	1.208 (2)	C11—C12	1.457 (2)
O4—C11	1.209 (2)	C12—C17	1.394 (2)
C1—C2	1.371 (3)	C12—C13	1.405 (2)
C1—C6	1.382 (2)	C13—C14	1.396 (3)
C2—C3	1.363 (3)	C14—C15	1.361 (3)
C2—H2A	0.9300	C14—H14A	0.9300
C3—C4	1.387 (3)	C15—C16	1.376 (3)
C3—H3A	0.9300	C15—H15A	0.9300
C4—C5	1.370 (3)	C16—C17	1.358 (3)
C4—H4A	0.9300	C16—H16A	0.9300
C5—C6	1.393 (3)	C17—H17A	0.9300
C5—H5A	0.9300		
			
C13—N1—H1A	119.3 (14)	O3—C9—C10	122.21 (16)
C13—N1—H1B	117.8 (16)	C8—C9—C10	115.14 (15)
H1A—N1—H1B	118 (2)	O2—C10—C9	111.46 (14)
C1—O1—C8	105.72 (13)	O2—C10—H10A	109.3
C11—O2—C10	115.04 (13)	C9—C10—H10A	109.3
C2—C1—O1	125.63 (17)	O2—C10—H10B	109.3
C2—C1—C6	124.29 (18)	C9—C10—H10B	109.3
O1—C1—C6	110.08 (15)	H10A—C10—H10B	108.0
C3—C2—C1	115.8 (2)	O4—C11—O2	121.09 (16)
C3—C2—H2A	122.1	O4—C11—C12	126.02 (15)
C1—C2—H2A	122.1	O2—C11—C12	112.89 (14)
C2—C3—C4	122.1 (2)	C17—C12—C13	118.93 (16)
C2—C3—H3A	119.0	C17—C12—C11	120.32 (15)
C4—C3—H3A	119.0	C13—C12—C11	120.72 (15)
C5—C4—C3	121.3 (2)	N1—C13—C14	119.72 (17)
C5—C4—H4A	119.3	N1—C13—C12	122.51 (17)
C3—C4—H4A	119.3	C14—C13—C12	117.76 (16)
C4—C5—C6	117.9 (2)	C15—C14—C13	121.56 (18)
C4—C5—H5A	121.1	C15—C14—H14A	119.2
C6—C5—H5A	121.1	C13—C14—H14A	119.2
C1—C6—C5	118.65 (17)	C14—C15—C16	120.74 (19)
C1—C6—C7	105.84 (15)	C14—C15—H15A	119.6
C5—C6—C7	135.50 (18)	C16—C15—H15A	119.6
C8—C7—C6	106.96 (16)	C17—C16—C15	119.00 (18)
C8—C7—H7A	126.5	C17—C16—H16A	120.5
C6—C7—H7A	126.5	C15—C16—H16A	120.5
C7—C8—O1	111.38 (15)	C16—C17—C12	121.99 (18)
C7—C8—C9	132.36 (17)	C16—C17—H17A	119.0
O1—C8—C9	116.19 (14)	C12—C17—H17A	119.0
O3—C9—C8	122.65 (17)		
			
C8—O1—C1—C2	179.91 (16)	O1—C8—C9—C10	177.70 (13)
C8—O1—C1—C6	−0.32 (16)	C11—O2—C10—C9	−70.85 (18)
O1—C1—C2—C3	179.33 (15)	O3—C9—C10—O2	−13.3 (2)
C6—C1—C2—C3	−0.4 (3)	C8—C9—C10—O2	166.80 (13)
C1—C2—C3—C4	−0.5 (3)	C10—O2—C11—O4	−0.3 (2)
C2—C3—C4—C5	1.0 (3)	C10—O2—C11—C12	179.52 (14)
C3—C4—C5—C6	−0.5 (3)	O4—C11—C12—C17	−175.17 (17)
C2—C1—C6—C5	0.8 (3)	O2—C11—C12—C17	5.1 (2)
O1—C1—C6—C5	−178.95 (14)	O4—C11—C12—C13	6.8 (3)
C2—C1—C6—C7	−179.49 (16)	O2—C11—C12—C13	−172.97 (14)
O1—C1—C6—C7	0.74 (17)	C17—C12—C13—N1	176.84 (17)
C4—C5—C6—C1	−0.3 (2)	C11—C12—C13—N1	−5.1 (2)
C4—C5—C6—C7	−179.90 (18)	C17—C12—C13—C14	−1.8 (2)
C1—C6—C7—C8	−0.87 (18)	C11—C12—C13—C14	176.29 (15)
C5—C6—C7—C8	178.74 (18)	N1—C13—C14—C15	−177.67 (18)
C6—C7—C8—O1	0.71 (18)	C12—C13—C14—C15	1.0 (3)
C6—C7—C8—C9	−176.21 (16)	C13—C14—C15—C16	0.6 (3)
C1—O1—C8—C7	−0.26 (17)	C14—C15—C16—C17	−1.3 (3)
C1—O1—C8—C9	177.21 (13)	C15—C16—C17—C12	0.4 (3)
C7—C8—C9—O3	174.59 (17)	C13—C12—C17—C16	1.1 (3)
O1—C8—C9—O3	−2.2 (2)	C11—C12—C17—C16	−176.94 (17)
C7—C8—C9—C10	−5.5 (3)		

### (II) 2-(1H-1-Benzofuran-2-yl)-2-oxoethyl 2-aminobenzoate Hydrogen-bond geometry (Å, º)

**Table:**

*D*—H···*A*	*D*—H	H···*A*	*D*···*A*	*D*—H···*A*
N1—H1*A*···O4	0.91 (2)	2.05 (2)	2.700 (3)	127.7 (18)
N1—H1*A*···O4^i^	0.91 (2)	2.49 (2)	3.246 (2)	141.4 (18)
C10—H10*A*···O3^ii^	0.97	2.50	3.444 (2)	165
C17—H17*A*···O2	0.93	2.35	2.687 (2)	101

Symmetry codes: (i) −*x*+1, −*y*+1, −*z*+1; (ii) *x*−1, *y*, *z*.
